# Effects of Urbanization on Ventral Patch Size and Phenotypic Correlates of Patch Expression in Male Western Fence Lizards ( *Sceloporus occidentalis* )

**DOI:** 10.1002/ece3.70915

**Published:** 2025-01-28

**Authors:** Breanna J. Putman, Bayley Stevens, Nina A. Fresco, Emily R. Urquidi

**Affiliations:** ^1^ Department of Biology California State University, San Bernardino California; ^2^ Department of Biological Sciences California State University Long Beach California

**Keywords:** colorful ornaments, glucocorticoids, phrynosomatidae, sexual signal, steroid hormones, ticks

## Abstract

In some animals, males use colorful ornaments or badges to visually communicate with conspecifics. These traits can be condition‐dependent, suggesting that environmental changes could impact the intensity of male sexual signals. Drastic habitat changes caused by urbanization can act as physiological stressors, potentially affecting male signaling traits through changes to condition or immune function. Here, we quantified the effects of urbanization on ventral patch size and correlates of patch expression, namely body size, body condition, corticosterone concentrations, and ectoparasites in male Western Fence Lizards ( *Sceloporus occidentalis* ). We compared three aspects of male ventral color patches between urban and natural populations: area of the throat patch, total area of the paired belly patches, and total area of the black borders of the belly patches. All three area measurements across both habitat types were positively related to body size, and total belly patch area was positively related to body condition, indicating that these traits may signal male competitive ability and/or quality. Males from urban populations had larger throat patches than those from natural populations after controlling for body size. This difference in patch size was associated with a difference in probability of ectoparasite infection, but not with differences in corticosterone concentrations or body condition between urban and natural populations. Our results may indicate an effect of urbanization on immune function affecting male patch expression, although this idea remains untested. Overall, we show that urbanization can impact male sexual traits, which may have repercussions for visual communication in urban environments.

## Graphical abstract

Urbanization affects male patch size and ectoparasite infections in western fence lizards. Patch expression and infections may be associated with each other as males with fewer immune challenges may be able to develop larger patches. Patch size also positively correlated with body size and body condition, indicating male fighting ability and/or quality.

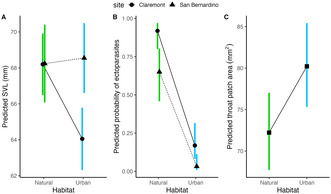

## 1 Introduction

Most of Earth's land has been impacted by humans (Ellis and Ramankutty 2008; Riggio et al. 2020). Urbanization, one of the most drastic forms of human impact, involves the process of building human infrastructure on once natural or rural habitats, and an associated increase in human population density (Moll et al. 2019). Urban environments pose challenges and opportunities for animals due to changes in food resources, shelter, predators, parasites, diseases, and sensory stimuli (Alberti et al. 2017; Lowry, Lill, and Wong 2013). Such habitat changes can act as acute or chronic stressors affecting the condition of animals. For instance, stressors associated with urbanization can increase stress hormones or glucocorticoid (GC) levels (Grunst et al. 2020; Iglesias‐Carrasco et al. 2020), which reallocate energy away from reproduction and reduce certain aspects of the immune system (Angelier and Wingfield 2012). Animals with higher circulating levels of GCs can be in worse body condition, potentially affecting development and reproduction (DeSimone et al. 2020; Malisch et al. 2020; Moore et al. 2000; Rensel, Wilcoxen, and Schoech 2011). In addition, changes to the prevalence and intensity of parasitic infections in urban habitats may be associated with shifts in immune function. Some animals are released from parasites in urban areas (Geue and Partecke 2008; Putman et al. 2021), whereas others may experience increased immune challenges due to higher levels of parasitic infections (Bonier et al. 2007; Jiménez‐Peñuela et al. 2023, 2019). Overall, such physiological responses could ultimately influence the fitness of urban‐dwelling animals (Angelier and Wingfield 2012; Bonier et al. 2007; Lucas and French 2012; Romero and Wilkelski 2001).

Males of many species use colorful badges or ornaments to indicate fighting ability to competitors and/or reproductive quality to potential mates (Emlen 2001; Houde 2001). These traits are often condition‐dependent—associating with immune function, body condition, and GC concentrations—allowing receivers to accurately assess male quality (Husak and Moore 2008; Leary and Baugh 2020). Thus, environmental stressors that affect an animal's condition can impact male sexual signals (Buchanan 2000). Because male sexual traits are often condition‐dependent, there is potential for urbanization to affect their expression or intensity (reviewed in Heinen‐Kay, Kay, and Zuk 2021). For example, carotenoid‐based male coloration, a classic condition‐dependent signal in many birds, can be affected by urbanization, with urban males exhibiting less colorful plumage than their natural counterparts (Giraudeau et al. 2018, Giraudeau, Chavez, Toomey, and McGraw, 2015; Grunst, Grunst, Pinxten, Bervoets, and Eens, 2020). Differences in male coloration between urban and non‐urban populations may suggest different types of diets (males acquire carotenoid pigments from foods) or exposure to more diseases and/or stress in urban habitats as carotenoids also have antioxidant abilities and contribute to immune function (Chew 1993; Costantini and Møller 2008; Tan et al. 2020; Weaver et al. 2018). Although there are documented effects of urbanization on condition‐dependent visual signals, most of this work has been narrowly focused on birds with little research into other taxonomic groups (Heinen‐Kay, Kay, and Zuk 2021).

Lizards have been studied extensively regarding the development, evolution, maintenance, and plasticity of male sexual visual signals (Chen et al. 2012; Macedonia, Clark, and Tamasi 2014; Ng et al. 2013; Ord, Stamps, and Losos 2010; Ossip‐Drahos et al. 2016). Males across taxa use colors, along with behavioral displays, for intraspecific communication, and these traits can exhibit population‐level variation based on habitat characteristics (Badiane et al. 2022; Leal and Fleishman 2004). The effects of urbanization on lizard phenotypic traits are increasingly being documented (Putman and Tippie 2020) and there is some evidence that the expression of male sexual signals is altered in urban habitats. Urban male rock agamas ( *Psammophilus dorsalis* ) have duller and paler colors during staged social encounters and slower rates of color change (Batabyal and Thaker 2017), urban male anoles ( *Anolis sagrei* ) have higher dewlap signaling rates (Stroud et al. 2019), and urban *Sceloporus torquatus* lizards have bluer ventral patches (González‐Morales et al. 2024) compared to males from non‐urban areas. Yet, to our knowledge, few other studies have quantified shifts in male sexual signals in lizards despite the widespread focus of this work within non‐urban populations. Lizards in the genus *Sceloporus* (commonly known as spiny lizards) are an ideal taxon for this type of research as the evolution and expression of male visual signals has been well‐studied (detailed below) and they occur across a range of habitats in North America from urban to rural areas.

*Sceloporus* lizards have males (and sometimes females) with colored patches on the throat and abdominal regions that are displayed via stereotypical posturing during conspecific interactions (Carpenter 1978). Patch size is thought to primarily signal male fighting ability to rival males because many studies show a positive association between patch size and body size (Langkilde and Boronow 2010; Ossip‐Drahos et al. 2018; Roberts, McElroy, and McBrayer 2024; Zúñiga‐Vega et al. 2021), and *S. undulatus* males modify their responses toward intruders with experimentally manipulated patch areas (Ossip‐Drahos et al. 2018). Patch components can also be condition‐dependent, for instance prior work has shown patch size to positively correlate with body condition (Roberts, McElroy, and McBrayer 2024; Zúñiga‐Vega et al. 2021) and immune response (Assis et al. 2021), and to negatively correlate with heterophil: lymphocyte ratios (an indicator of physiological stress) (Zúñiga‐Vega et al. 2021). Testosterone can increase both the size and color saturation of *Sceloprous* ventral patches (Cox et al. 2005; Pollock et al. 2017; Robinson, Lance, and Gifford 2021; Robinson and Gifford 2019), whereas corticosterone (the main GC in reptiles) can negatively affect these traits (Argaez et al. 2021; Assis et al. 2021; Calisi and Hews 2007). Both testosterone and corticosterone have immunosuppressive effects (Foo et al. 2017; Webster Marketon and Glaser 2008), implying that only healthy individuals can produce high‐quality signaling patches, an idea known as the immunocompetence handicap hypothesis (Folstad and Karter 1992). The immunosuppressive effects of these hormones may reduce lizards' defense against parasites (Owen, Nelson, and Clayton 2010); indeed, in lizards, high levels of plasma testosterone associate with higher parasite loads (Pollock, Vredevoe, and Taylor 2012), which in turn can positively correlate with the intensity of patch coloration (Megía‐Palma et al. 2018; Salvador et al. 1996; Zúñiga‐Vega et al. 2021) or patch size (Roberts, McElroy, and McBrayer 2024; Salvador et al. 1996). Additional work suggests that lizards with low levels of parasitic infections may be able to allocate more resources to ventral patch expression, resulting in more intense coloration (González‐Morales et al. 2024; Rivera‐Rea et al. 2024). Thus, patch attributes may vary based on the signaler's size, condition, and immunological condition, allowing receivers to accurately assess male quality.

*Sceloporus* patch attributes may also vary between populations (García‐Rosales et al. 2017; Langkilde and Boronow 2010). For instance, the condition‐dependent female ornaments in *Sceloporus virgatus* are larger and more orange in unburned habitats than in habitats that recently experienced wildfire (Weiss and Brower 2021), *Sceloporus occidentalis* populations with lower baseline corticosterone levels have darker ventral areas, but also higher mite loads (Seddon and Hews 2017), and the relationship between mite load and badge area differs among habitats in *Sceloporus woodi* (Roberts, McElroy, and McBrayer 2024). A recent study also found that *Sceloporus torquatus* lizards living in urban habitats have lower parasite loads, lower immune responses, and more intense (bluer) coloration of male ventral patches (González‐Morales et al. 2024). Collectively these studies suggest that habitat‐specific environmental conditions affecting the body size, health and/or condition of lizards, or affecting the signaling environment can modify visual signaling traits.

Here, we sought to determine whether urbanization affects the body size, condition, GC levels (measured as baseline corticosterone), ectoparasite infections, and ventral patch sizes of male Western Fence Lizards ( *Sceloporus occidentalis* ). We predicted that urban lizards would be smaller, in worse condition, and have higher GC levels than their natural counterparts based on prior research (Lucas and French 2012; Putman et al. 2024). Conversely, urban lizards were expected to have fewer ectoparasites than those in natural habitats due to a reduction in available hosts necessary for a complex life cycle and the widespread use of pesticides and anti‐tick medication by people in urban areas (Putman et al. 2021). If urban lizards are smaller, in worse condition, and/or have higher levels of circulating stress hormones, they may also express smaller ventral patches than their natural counterparts. However, patch size may also be associated with immunological challenges (i.e., intensity of ectoparasite infections). If urban lizards are released from ectoparasite infections, they may have larger patches because they can re‐allocate energy from the immune system to color expression (González‐Morales et al. 2024). We also looked for relationships between patch size and these other traits to determine whether patch size indicates aspects of male quality in this species and whether these relationships differ between urban and non‐urban populations.

## 2 Methods

### 2.1 Study Species and Sites

Western Fence Lizards ( *Sceloporus occidentalis* ) were measured as part of ongoing research at urban and natural habitats in Southern California. Urban habitats consisted of two college campuses: California State University, San Bernardino (CSUSB; 34.183253, −117.323654) and the Claremont Colleges (34.101650, −117.711519). These two sites were similar in structure with large campus buildings interspersed by walkways, manicured lawns, and landscaping. Natural habitats were non‐urban sites approximately 1 km away from the urban sites that mostly consisted of native oak, sage scrub, and chaparral vegetation. In San Bernardino, the natural habitat was located across from the CSUSB campus at the base of the San Bernardino Mountains (34.191438, −117.312396). In Claremont, the natural habitat was located at the Robert J. Bernard Biological Field Station (part of the Claremont Colleges; 34.109088, −117.711791). The distance between urban and non‐urban populations is large enough to ensure that individual lizards are not directly migrating between the urban and non‐urban sites based on the average dispersal distance and home range size of this species (Davis and Ford 1983; Massot et al. 2003), but gene flow likely occurs between them.

### 2.2 Lizard Capture and Processing

We captured 136 male lizards (minimum snout‐vent‐length of 51 mm) in 2020–2022 from April to July between the hours of 0900 and 1600. We caught lizards with a loop of fishing line attached to the end of a telescoping fishing pole. For each lizard, we quantified snout‐vent‐length (SVL), a measure of body size, using a ruler to measure from the tip of the lizard's snout to the middle of the cloaca or vent. We used a digital scale or spring scale to quantify body mass (both rounded to the nearest tenth), and computed the scaled mass index of body condition from mass and the linear body measurements of SVL (Peig and Green 2009). We quantified ectoparasite presence on each lizard by determining whether or not ticks and/or mites were visibly present. We also counted the number of ticks visible on lizards. Mites were unable to be reliably counted because of their small size. We balanced data collection among the four sites so that the average date of capture was the same between natural and urban habitats.

Starting in 2021, we collected blood samples from 70 individuals immediately after capture to estimate baseline levels of corticosterone (CORT) concentrations. Blood samples were only taken from 0900 to 1300 to restrict variation in CORT due to diel fluctuations. Lizards were bled within 4 min of starting the capture attempt, calculated using a handheld stopwatch. This is a reasonable amount of time before CORT levels increase due to human‐induced stress (Putman et al. 2024; Tylan et al. 2020). The blood was extracted from the post‐orbital sinus using a heparinized microcapillary tube, and samples were kept on ice in microcentrifuge tubes until we returned to the lab. After capture and during blood extraction, we took the lizard's body temperature using a laser infrared thermometer (ennoLogic, et650D). In the lab, blood samples were spun down at 3.0 *rcf* for 10 min. We used a Hamilton syringe to collect the plasma, which was then transferred to a cryotube for storage in a −80°C freezer. We quantified CORT levels using an ELISA kit (Enzo Life Sciences, ADI‐901‐097). This kit has been optimized and validated previously for use in Western Fence Lizards by our lab (Putman et al. 2024). Plasma samples were diluted at a 1:40 ratio with a 1% concentration (of raw plasma volume) of steroid displacement buffer and tested on a 96 well plate in duplicate. Using a plate reader, we read the samples at 405 nm after a 60‐min incubation period. We calculated the concentration of CORT using the absorbance readings by taking the average of the duplicate samples. The minimum level of detection for this kit is 0.032 ng/mL, so any samples that were lower than this concentration were assigned this minimum concentration (*N* = 12 of 70). Blood samples were randomized on plates with samples from another study that were collected concurrently. Plates had between 6 and 14 samples from this study (average = 8.8) with a relatively even distribution of samples from natural habitats and urban habitats on each plate (the average natural to urban habitat ratio was 0.86 with a higher N of urban habitat samples compared to natural habitat samples). Intraassay coefficient of variation (CV) ranged from 10% to 26% with an average of 17% (*N* = 8 plates). The interassay CV was 16% (with 4 CORT standards on each plate).

### 2.3 Quantification of Patch Sizes

**FIGURE 1:**
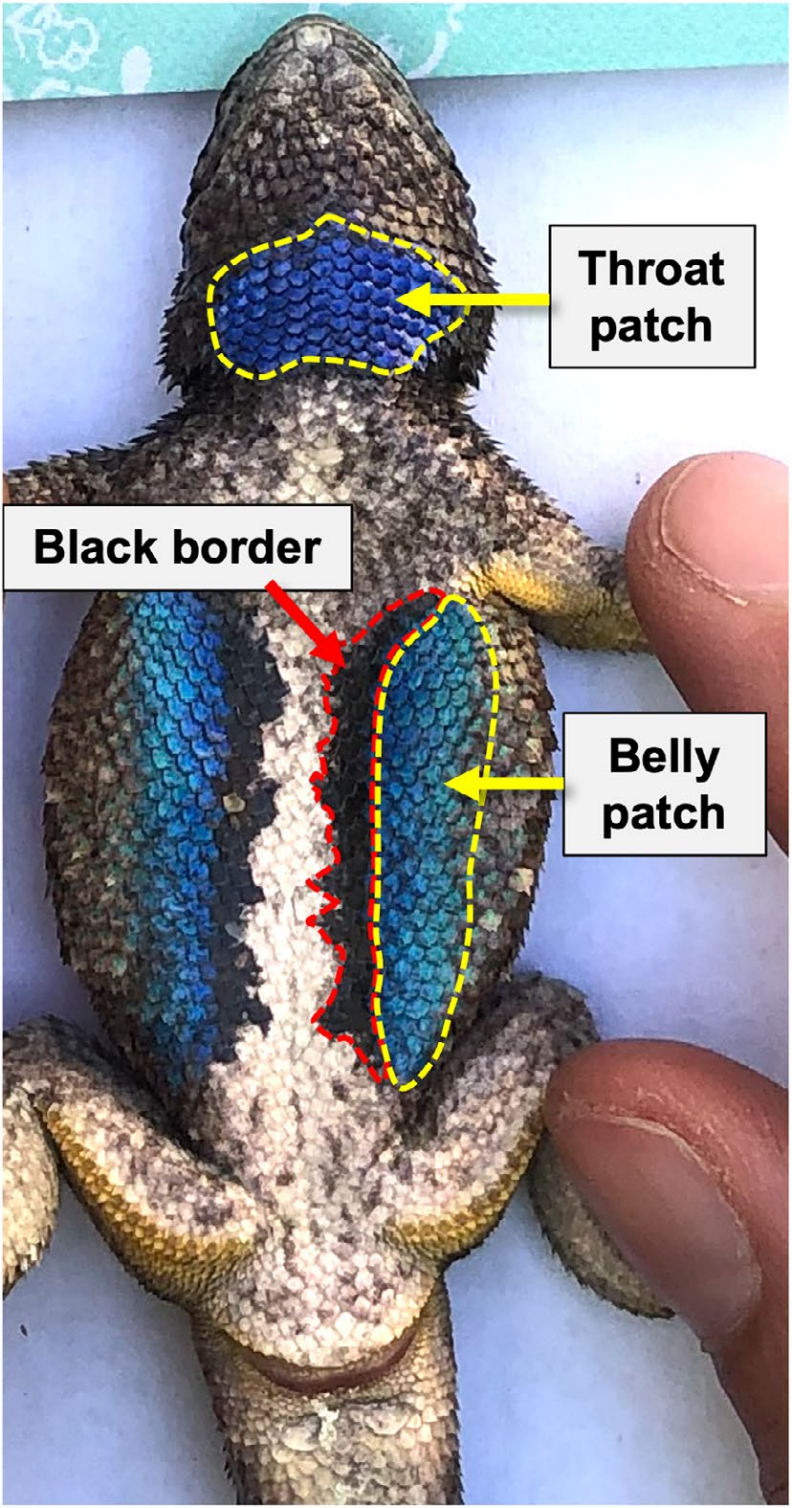
Photo of an individual lizard used in this study showing the typical ventral patches of male *Sceloporus occidentalis* .

We used standard methods to photograph the lizards' ventral patches using an Olympus Tough TG‐6 digital camera (Robinson, Lance, and Gifford 2021; Zúñiga‐Vega et al. 2021). To take the pictures, the lizard was laid flat on its dorsal side on blank white paper with a ruler to indicate a standard size. A small tripod was placed above the lizard with the digital camera attached (approx. 25 cm above the lizard), and the camera was leveled using a built‐in level to ensure it was exactly parallel to the lizard. Pictures were taken without flash in the field in complete shade to reduce variation in lighting or in the lab under constant lighting conditions. We manually reviewed each photo to verify that all scales that made up the patch areas were visible. The pictures were analyzed using ImageJ with the source location of the lizard (e.g., urban or non‐urban) blind to the reviewer. The ruler in each photo was used to set the scale in ImageJ. Image SVL was quantified using the line tool by drawing a line from the tip of the lizard's snout to the center of the cloaca. The areas of each colored patch (blue throat and two lateral belly patches), along with the areas of the two black borders of the belly patches were measured using the polygon tool (Figure 1). The areas of the right and left sides of the blue belly patches and their borders were added together to calculate total area for these regions.

Two persons reviewed the photos, but did not review the same photos. Within reviewers, they achieved coefficients of variation less than or equal to 5% in SVL and patch area measurements (Zúñiga‐Vega et al. 2021). To determine whether the two reviewers were consistent in their quantifications (between reviewer consistency), they were compared using a training dataset of 19 lizard photos from which they measured all traits listed above. We used paired *t*‐tests to determine whether the mean difference in the reviewers' scores was different from zero and calculated the intraclass correlation coefficient as a measure of reviewer agreement in responses (Bland and Altman 2010; Fleiss and Cohen 1973). Overall, reviewers mostly exhibited near perfect agreement in calculations (Table A1) (Sung, Bhan, and Vernon 2002), meaning that they consistently coded lizard traits in the same way (i.e., lizards with larger SVLs were consistently coded as having larger SVLs and lizards with larger patches were coded as having larger patches). However, based on the paired t‐tests one reviewer tended to calculate larger areas for the blue belly patches and the throat patch on the same lizards than the other reviewer (Table A1). Thus, for our final statistical models, we included reviewer ID as an independent variable to account for differences between the two reviewers.

### 2.4 Statistical Analyses

All statistical analyses were done in R (v 4.2.3) (R Core Team 2023) and alpha was set to 0.05. When applicable, we conducted pairwise comparisons using the emmeans package (Lenth et al. 2023) and *p* values were adjusted by the false discovery rate method. Assumptions of models were met based on examination of residual plots.

We determined whether adult male fence lizards from urban habitats differed in body size (SVL), body condition (as scaled mass index), ln‐transformed values of baseline CORT, and/or ectoparasite presence compared to those from natural habitats. For these analyses we used linear models with habitat (urban vs. natural) and site (Claremont vs. San Bernardino) and their interaction as independent variables. We included site in all models to account for potential differences in the urban campus environments and natural habitats between the two locations. For the model on baseline CORT, we looked at whether other variables importantly affected CORT concentrations, as has been shown in the past (Putman et al. 2024), but found none to be significant (time to bleed: *F*_1,65_ = 1.480, *p* = 0.228; body size: *F*_1,65_ = 0.252, *p* = 0.618; body condition: *F*_1,65_ = 0.899, *p* = 0.347; lizard body temperature: *F*_1,65_ = 2.883, *p* = 0.094, or capture date: *F*_1,65_ = 3.299, *p* = 0.074). For ectoparasite presence, we used a logistic regression model examining the effects of habitat, site, and their interaction on probability of ectoparasite infection. For all models, if the interaction term was non‐significant, it was removed from the model and only the main effects were assessed.

We used linear models to look for the effect of urbanization (coded as habitat: urban or natural) on male patch sizes. In each model, we evaluated the effects of habitat, site, reviewer ID, and image SVL (log‐transformed) on the log‐transformed size of each signaling patch area (blue throat, blue belly, and black border) for a total of three separate models. We used image SVL in these models because it was measured the same way as the patch areas in ImageJ and we had high levels of repeatability and consistency between reviewers for this trait. Image SVL was also highly correlated with the SVL values taken by hand (Pearson's *r* = 0.94, *t* = 30.3, *p* < 0.001). We also determined whether male ornament expression (i.e., patch size) was correlated with variation in body condition (as scaled mass index), number of ticks, and baseline CORT concentrations through inclusion of these numerical covariates in the models. To avoid over‐fitting models, these covariates were only included when they improved model fit, which was determined through likelihood ratio tests (Table A2). We looked for significant interactions between the independent variables in all models, and if the interaction terms were non‐significant, they were removed and only the main effects were assessed.

## 3 Results

**FIGURE 2:**
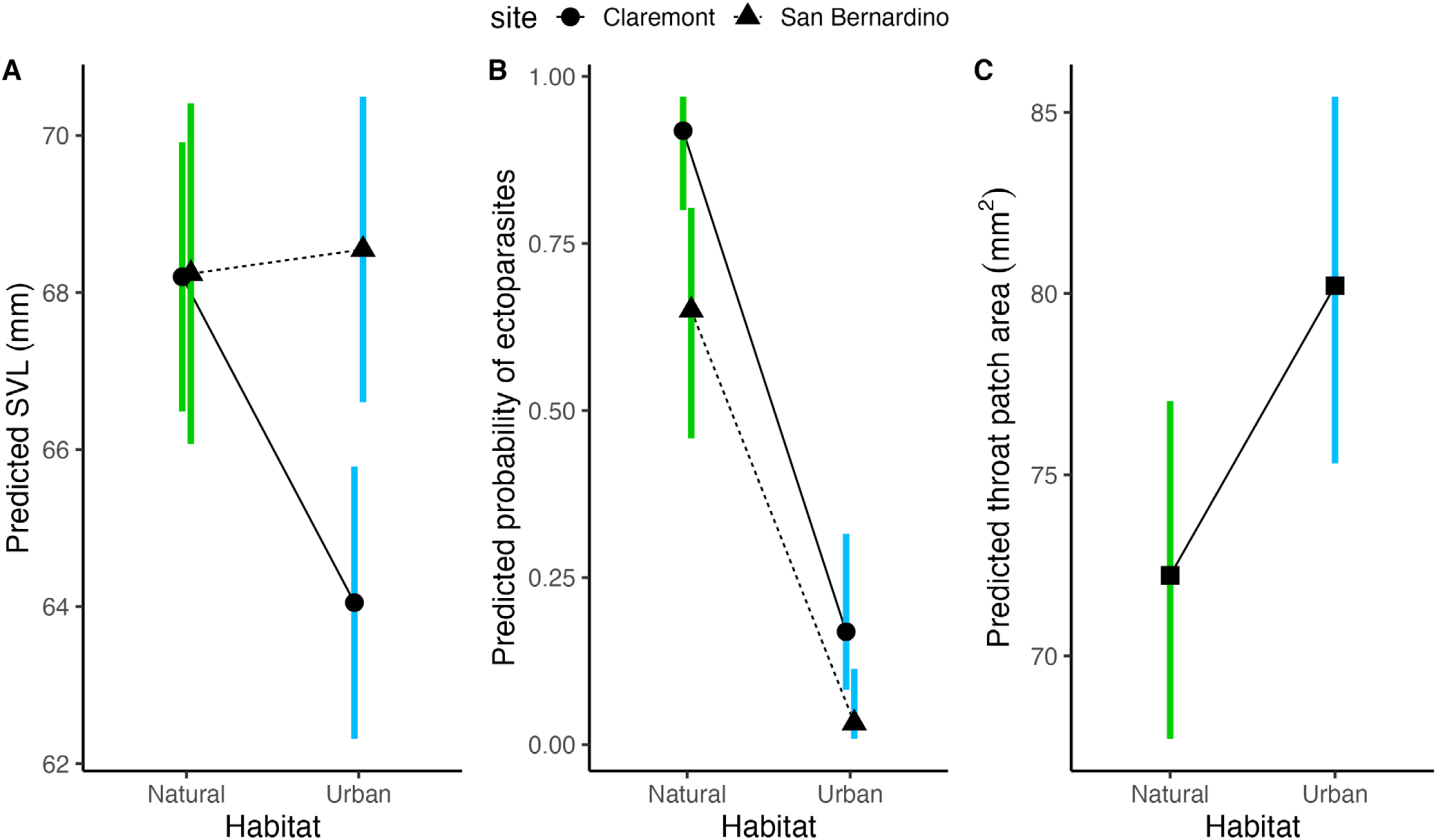
Estimated marginal means and 95% confidence intervals from linear or logistic models predicting the effect of habitat along with other covariates on (A) lizard body size measured as SVL, (B) probability of ectoparasite infection, and (C) throat patch area.

We quantified body size of 135 male fence lizards (*N* = 65 from natural habitat and *N* = 70 from urban habitat) and body condition of 133 male fence lizards (*N* = 63 from natural habitat and *N* = 70 from urban habitat). The effect of urbanization on body size was dependent on site (estimate = 4.46 ± 1.92, *t =* 2.32, *p* = 0.022, Figure 2A). Urban lizards were significantly smaller compared to natural lizards in Claremont (estimate = 4.15 ± 1.23, *t =* 3.37, *p =* 0.001), but there was no difference in lizard body size between the two San Bernardino populations (estimate = −0.308 ± 1.47, *t = −*0.210, *p =* 0.834). Neither habitat type (estimate = 0.172 ± 0.212, *t* = 0.814, *p* = 0.417) nor site (estimate = −0.142 ± 0.215, *t* = −0.660, *p* = 0.511) affected lizard body condition. We determined ectoparasite presence of 125 male fence lizards (*N* = 65 from natural habitat and *N* = 60 from urban habitat). Lizards in urban habitats were less likely to have ectoparasites than those in natural habitats (estimate = −4.02 ± 0.628, *Z* = −6.40, *p* < 0.001, Figure 2B), and lizards in Claremont had a higher probability of infection than lizards in San Bernardino (effect of site: estimate = 1.80 ± 0.621, *Z* = 2.91, *p* = 0.004). In urban habitats, only 11% of lizards had ectoparasites, while 82% of lizards caught in natural areas had ectoparasites. At the San Bernardino site, no urban lizards had ectoparasites. Finally, we quantified baseline CORT of 70 individuals (*N* = 31 from natural habitat and *N* = 39 from urban habitat). There was no effect of urbanization (estimate = 0.291 ± 0.218, *t* = 1.340, *p* = 0.185) or site (estimate = 0.032 ± 0.216, *t* = 0.147, *p* = 0.883) on baseline CORT concentrations.

**FIGURE 3:**
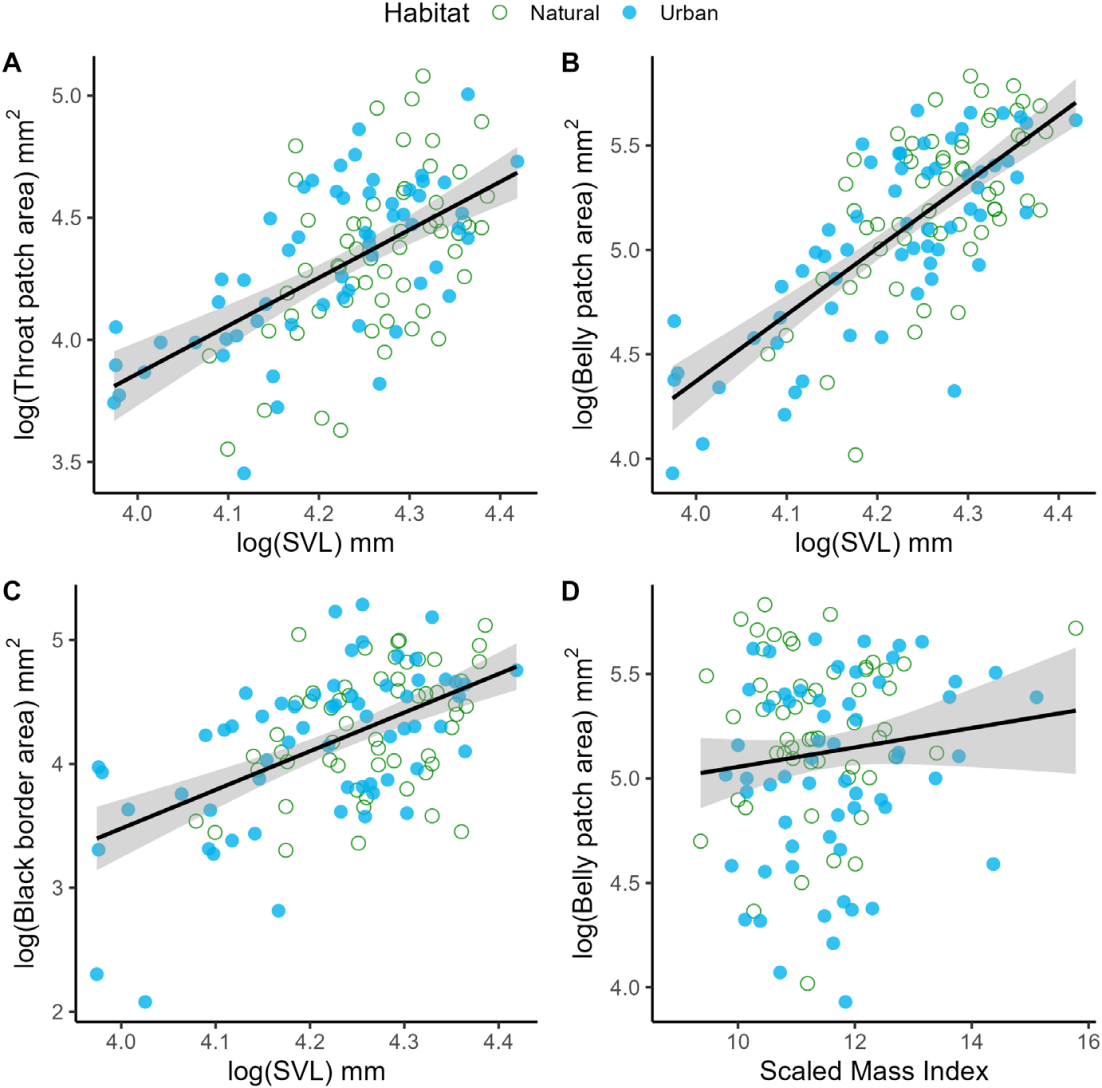
Relationships between body size and male signaling traits: (A) blue throat patch area, (B) blue belly patch area, and (C) black border area; and (D) the relationship between lizard body condition, estimated as the scaled mass index, and belly patch area in male western fence lizards.

In total, we photographed the ventral patches of 58 male fence lizards from natural habitats (26 reviewed by B.S. and 32 reviewed by N.F.) and 62 lizards from urban habitats (30 reviewed by B.S. and 32 reviewed by N.F.). Urban lizards had significantly larger throat patches compared to their natural counterparts after controlling for body size (estimate ± SE = 0.105 ± 0.046, *t* = 2.260, *p* = 0.026, Figure 2C), but there was no difference between urban and natural populations in the size of the blue belly patches (estimate ± SE = −0.012 ± 0.050, *t* = −0.248, *p* = 0.805) or their black borders (estimate ± SE = 0.059 ± 0.091, *t* = 0.652, *p* = 0.515). All patch areas were positively related to lizard body size (throat: estimate ± SE = 2.394 ± 0.250, *t* = 9.573, *p* < 0.001; belly: estimate ± SE = 3.646 ± 0.270, *t* = 13.504, *p* < 0.001; border: estimate ± SE = 3.519 ± 0.489, *t* = 7.198, *p* < 0.001; Figure 3A–C). We also found that blue belly patch size increased with lizard body condition (estimate ± SE = 0.043 ± 0.021, *t* = 2.065, *p* = 0.041, Figure 3D). We did not find other relationships between patch sizes and condition‐associated traits (e.g., number of ticks or CORT; Table A2).

Reviewers differed in area calculations for the blue throat and belly patches (throat: estimate ± SE = 0.278 ± 0.046, *t* = 5.991, *p* < 0.001; belly: estimate ± SE = 0.292 ± 0.050, *t* = 5.863, *p* < 0.001), but not for the black borders (estimate ± SE = −0.053 ± 0.091, *t* = −0.581, *p* = 0.563) in concordance with our training dataset comparisons (Table A1). There was no effect of site on throat patch area (estimate ± SE = 0.065 ± 0.047, *t* = 1.382, *p* = 0.170) or belly patch area (estimate ± SE = −0.041 ± 0.050, *t* = −0.818, *p* = 0.415), but lizards in Claremont had larger black borders than those in San Bernardino (estimate ± SE = 0.317 ± 0.092, *t* = 3.434, *p* = 0.001).

## 4 Discussion

In western fence lizards, urbanization is associated with shifts in the size of male signaling patches and in ectoparasite infections based on the results of our study. After controlling for body size, urban lizards expressed larger throat patches than their natural counterparts and were nearly released from ectoparasitic infections (at one urban site, no lizards had ectoparasites). Urbanization did not have an effect on body condition or corticosterone concentrations, and only affected lizard body size at one site with urban lizards being smaller than those in the natural habitat. Taken together, these results suggest that urbanization may have minimal negative impacts on the health or condition of lizards at these sites; on the contrary, urban lizards may have higher levels of immunocompetence or fewer immunological challenges associated with reduced ectoparasite infections compared to their natural counterparts. This may allow them to produce relatively larger throat patches for sexual signaling.

Ectoparasitic infections were expected to be lower in urban populations due to disruptions to parasite life cycles and the widespread use of pesticides and anti‐tick medications by people in urban areas. A pattern of lower ectoparasite infections in urban populations has been supported widely in lizards (Carbayo, Martín, and Civantos 2019; González‐Morales et al. 2024; Putman et al. 2021) and other taxa (DeVore, Shine, and Ducatez 2020; Heller et al. 2019; Wemer et al. 2021; Werner and Nunn 2020). It is possible the difference in parasitic infections is also due to different levels of immunocompetence between urban and natural lizard populations. Urban lizards may be exposed to different environmental conditions (e.g., higher nutritional diets or elevated pathogen pressures) that lead to an increased immune response (Minias 2023; Ruiz et al. 2010), thereby lowering ectoparasitic loads. Thus, the larger throat patch areas of urban males could indicate superior immune systems (i.e., good genes) as predicted by the Humilton‐Zuk hypothesis (Hamilton and Zuk 1982). In support of this, there is a positive correlation between male badge area and saturation and immune response in *Sceloporus undulatus* (Assis et al. 2021), a positive correlation between throat patch luminance and immune response in *Sceloporus torquatus* (Rivera‐Rea et al. 2024), and a negative correlation between patch saturation or brightness and number of heterophils (indicating low infection rates) in four *Sceloporus* species (Zúñiga‐Vega et al. 2021). However, these studies mostly associate immune function with patch coloration and not patch size, which may reflect a different aspect of male quality (Zúñiga‐Vega et al. 2021). Furthermore, many studies show urban animals to have higher disease and parasitic infection rates (Bradley and Altizer 2007; Murray et al. 2019) and lower immune responses (González‐Morales et al. 2024) than their natural counterparts, so further work will need to quantify the specific contributions of the immune system on lizard patch attributes in urban environments.

Conversely, the immunocompetence handicap hypothesis states that due to the immunosuppressive effects of testosterone (one of the main hormones regulating male sexual traits), only males of high condition or quality can express the most exaggerated traits because they can resist infections (Folstad and Karter 1992). This hypothesis predicts that males with larger or more colorful signals should be the most vulnerable to ectoparasite infections. Indeed, in *Sceloporus* lizards, experimentally increasing testosterone leads to larger throat and belly patch areas (Cox et al. 2005; Salvador et al. 1996), but lizards with higher testosterone levels are also more susceptible to ectoparasites (Fuxjager et al. 2011; Pollock, Vredevoe, and Taylor 2012; Salvador et al. 1996). A pattern of higher ectoparasite abundance with increasing levels of testosterone has been widely reported in reptiles (Roberts, Buchanan, and Evans 2004). In habitats where ectoparasites are rare or non‐existent (like in our urban San Bernardino population), individuals may not need as robust of immune systems as those in natural habitats. Thus, males may be able to have higher circulating testosterone levels without experiencing the negative impacts of ectoparasites (Lanser, Vredevoe, and Kolluru 2021), or lower ectoparasite loads may allow lizards to allocate more resources to coloration, which is energetically costly to produce (Rivera‐Rea et al. 2024). In support of this, we found that urban lizards had a much lower probability of ectoparasitic infections and larger relative throat patch sizes than their natural counterparts. Another recent study also found urban *Sceloporus* lizards had lower parasite loads and more intense (blue) coloration compared to non‐urban lizards (González‐Morales et al. 2024). Furthermore, when we looked at the direct effect of ectoparasite load on patch size, we found a positive relationship between number of ticks and throat patch area, although this effect did not reach statistical significance (Table A2). Thus, our findings suggest that lizards may be able to develop larger relative throat patches in habitats where ectoparasite infections are rare (urban habitats), and throat patch area may indicate tolerance to ectoparasites in habitats where infections are more common (natural habitats). However, we did not quantify testosterone levels or look at other indicators of immune response and so these ideas are speculative and deserve testing. We found that populations do not differ in corticosterone concentrations, which could negatively influence the expression of male sexual traits and immune function (Assis et al. 2021; Calisi and Hews 2007; Lindsay et al. 2016). Thus, this is likely not the mechanism explaining the difference in throat patch size between urban and natural lizard populations.

Larger throat patch areas in urban lizards could also result from divergent sexual selection pressures between urban and non‐urban environments (Cronin et al. 2022), although it is not clear why this would only impact throat patch area and not the other patches. Selection on signal detectability in an altered environment (e.g., the sensory drive hypothesis) sometimes explains habitat differences in male patch attributes (Cummings and Endler 2018). Male sexual signaling colors in lizards can vary based on habitat; for example, different colors may be more detectable in low light versus high light environments (Badiane et al. 2022; Fleishman, Wadman, and Maximov 2020; Leal and Fleishman 2004). We did not measure aspects of the signaling environments in our study so we cannot determine whether larger throat patch areas may be more detectable in urban habitats than more natural ones. There is some support that male patch color can influence individual reproductive success, but generally, natural and sexual selection are thought to act weakly on male color signals in lizards (Ord et al. 2015; Robinson, Lance, and Gifford 2021). Further research should assess signal detectability of various patch attributes (size and color) in urban and non‐urban environments.

Across all populations, we found highly significant relationships between lizard body size and patch areas, and a significant relationship between body condition and the blue belly patch area. All patch areas measured increased with body size, and the area of the blue belly patch increased with body condition. This suggests that the size of male sexual signals indicates male fighting ability to competitors and/or quality to potential mates, corroborating prior research on the information encoded in *Sceloporus* ventral patches (Goodlett and Stephenson 2019; Ossip‐Drahos et al. 2018; Roberts, McElroy, and McBrayer 2024; Zúñiga‐Vega et al. 2021). These relationships were not different between urban and natural populations suggesting a conserved function that is not strongly affected by environmental conditions. Furthermore, the size of signaling patches did not associate with corticosterone concentrations, although our CV values for the corticosterone assays were relatively high, which could have hindered our ability to detect an effect. However, despite some prior work showing negative relationships between GCs and male colored ornaments in lizards (Argaez et al. 2021; Assis et al. 2021; Calisi and Hews 2007; San‐Jose and Fitze 2013), other studies have also failed to find significant relationships between these two traits (MacLeod, McCormick, and Langkilde 2019; Zúñiga‐Vega et al. 2021). Corticosterone may not be as important of a hormone as testosterone in regulating patch attributes in this group of lizards or may only influence male sexual traits during certain times of year or environmental conditions (Argaez et al. 2021; Seddon and Hews 2020).

We also found site specific effects on lizard traits. The effect of urbanization on lizard body size was only apparent at the Claremont site, and the black borders of the blue belly patches were also larger in Claremont lizards compared those in San Bernardino. These results may be explained by different environmental conditions between the two sites. For instance, San Bernardino is more inland and has lower tree cover than Claremont (Putman, unpublished data). In common lizards (*Zootoca vivipara*), male color signals are more saturated in forested habitats, suggesting that these sexual traits are modified for the signaling conditions of the specific environment (Badiane et al. 2022). The black borders of the belly patches likely help define the colored areas, increasing their conspicuousness to receivers (Bókony et al. 2003). Prior work has also found a positive relationship between male body condition and black border area, suggesting that this could be an additional signal of male quality (Zúñiga‐Vega et al. 2021). The black borders are the result of a layer of melanophores containing melanin that extend beyond a layer of iridophores that create the blue color of the ventral patches (Assis et al. 2021; Morrison, Rand, and Frost‐Mason 1995; Quinn and Hews 2003). The development of melanophores is dependent on androgens (e.g., testosterone) (Cox et al. 2005; Pollock et al. 2017), therefore, population differences in androgen levels, along with different signaling environments may explain the difference in black border size between Claremont and San Bernardino lizards.

In summary, we show that urbanization affects ectoparasite infections and male sexual signals in *Sceloporus occidentalis* lizards. Based on these results, urban lizards may have different immunological challenges or different levels of immunocompetence than their natural counterparts. Furthermore, differences in the relative throat patch size between habitats could suggest that different hormonal mechanisms or signaling environments affect this male sexual trait. Finally, we found support for the size of patch areas to signal male body size and condition to potential receivers, confirming prior research on the information encoded in *Sceloporus* ventral patches. Future work should quantify immune function, testosterone levels, the direct contributions of these traits to patch expression, and signal detectability in different habitats to understand the mechanism(s) leading to the differential throat patch sizes between urban and non‐urban populations.

## Author Contributions

**Breanna J. Putman:** conceptualization (lead), formal analysis (lead), funding acquisition (lead), supervision (lead), writing – original draft (lead). **Bayley Stevens:** data curation (equal), investigation (equal), writing – review and editing (equal). **Nina A. Fresco:** data curation (equal), investigation (equal), writing – review and editing (equal). **Emily R. Urquidi:** conceptualization (supporting), data curation (equal), formal analysis (supporting), investigation (lead), project administration (lead), writing – review and editing (equal).

## Conflicts of Interest

The authors declare no conflicts of interest.

## Data Availability

Data, metadata, and code are openly available on Dryad: https://doi.org/10.5061/dryad.8sf7m0d0k

## Appendix A

**TABLE A1:** Between reviewer consistency in quantifying lizard traits from photos, including the mean difference in scores between the two reviewers, the 95% confidence interval around the mean difference, the *t‐*value and associated *p* value from paired *t*‐tests, and the intraclass correlation coefficients (ICC). ICC classifications taken from Sung, Bhan, and Vernon (2002).

Lizard trait	Mean difference	95% CI	*t*	*p*	ICC	ICC classification
Image SVL	−0.14	−1.1, 0.82	0.33	0.751	0.99	Almost perfect agreement
Throat patch area	−6.69	−12.1, −1.2	2.58	**0.019**	0.92	Almost perfect agreement
Left blue belly patch area	−16.00	−20.9, −11.2	6.94	**< 0.001**	0.96	Almost perfect agreement
Right blue belly patch area	−17.00	−23.1, −10.9	5.84	**< 0.001**	0.96	Almost perfect agreement
Left black border area	−1.23	−6.7, 4.2	0.48	0.640	0.75	Substantial agreement
Right black border area	−1.08	−6.1, 3.9	0.45	0.655	0.93	Almost perfect agreement

**TABLE A2:** Results of likelihood ratio tests comparing linear models with and without predictor variables. The difference in log‐likelihoods between the models is compared with a chi‐squared distribution. All base models contained the predictors of habitat (urban vs. natural), site (Claremont vs. San Bernardino), reviewer ID, and Image SVL (log‐transformed). Predictors which reached statistical significance are bolded.

Dependent variable	Predictor	Likelihood base model	Likelihood base model + predictor	df	*χ* ^2^	*p*
Log (throat patch area)	Body condition	5.0861	5.4307	1	0.689	0.406
	CORT	6.1721	6.2195	1	0.095	0.758
	Ticks	2.4293	4.3191	1	3.780	0.052
Log (belly patch area)	**Body condition**	**−3.5134**	**−1.3065**	**1**	**4.414**	**0.036**
	CORT	−4.9291	−4.9274	1	0.003	0.954
	Ticks	−3.2835	−3.2588	1	0.049	0.824
Log (black border area)	Body condition	−70.132	−70.125	1	0.014	0.908
	CORT	−22.640	−22.564	1	0.153	0.695
	Ticks	−66.394	−66.378	1	0.030	0.862
